# Near‐Infrared Light‐Driven MXene/Liquid Crystal Elastomer Bimorph Membranes for Closed‐Loop Controlled Self‐Sensing Bionic Robots

**DOI:** 10.1002/advs.202307862

**Published:** 2023-11-20

**Authors:** Youwei Yang, Lingxian Meng, Juzhong Zhang, Yadong Gao, Zijuan Hao, Yang Liu, Mingjun Niu, Xiaomeng Zhang, Xuying Liu, Shuiren Liu

**Affiliations:** ^1^ School of Materials Science and Engineering Zhengzhou University Zhengzhou 450001 P. R. China; ^2^ School of Chemical Engineering Zhengzhou University Zhengzhou 450001 P. R. China

**Keywords:** biomechanics, closed‐loop, liquid crystalline elastomers, photothermal actuators, self‐sensing

## Abstract

More recently, soft actuators have evoked great interest in the next generation of soft robots. Despite significant progress, the majority of current soft actuators suffer from the lack of real‐time sensory feedback and self‐control functions, prohibiting their effective sensing and multitasking functions. Therefore, in this work, a near‐infrared‐driven bimorph membrane, with self‐sensing and feedback loop control functions, is produced by layer by layer (LBL) assembling MXene/PDDA (PM) onto liquid crystal elastomer (LCE) film. The versatile integration strategy successfully prevents the separation issues that arise from moduli mismatch between the sensing and the actuating layers, ultimately resulting in a stable and tightly bonded interface adhesion. As a result, the resultant membrane exhibited excellent mechanical toughness (tensile strengths equal to 16.3 MPa (||)), strong actuation properties (actuation stress equal to 1.56 MPa), and stable self‐sensing (gauge factor equal to 4.72) capabilities. When applying the near‐infrared (NIR) laser control, the system can perform grasping, traction, and crawling movements. Furthermore, the wing actuation and the closed‐loop controlled motion are demonstrated in combination with the insect microcontroller unit (MCU) models. The remote precision control and the self‐sensing capabilities of the soft actuator pave a way for complex and precise task modulation in the future.

## Graphical abstract

A light‐driven and self‐sensing intelligent bilayer soft actuator, the PM‐liquid crystal elastomer (LCE), built with LCE, MXene, and Poly(dimethyldiallylammonium chloride) (PDDA) is prepared through the layer‐by‐layer self‐assembly strategy; moreover, the high‐precision, self‐sensing and feedback loop control functions are realized. Furthermore, a PM‐LCE‐based closed‐loop control system is also demonstrated.

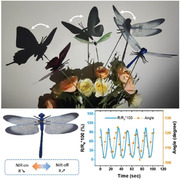

## 1 Introduction

The biological system has embedded sophisticated organisms that hold finely tuned and versatile regulatory processes, allowing them to effectively navigate and respond to changes in their surroundings. For instance, the auto‐capture of prey by Venus flytraps, the automatic closing of the human iris when being exposed to different lighting conditions, and the circadian rhythm of sunflower plants are examples of such complex systems.^[^ ^1^ ^]^ This rich source of inspiration provides designers and engineers the opportunity to create intelligent robots that possess similar precise and autonomous perception feedback and behavior regulation.

Different and enormous bioinspired smart materials and actuators, with synergistic environmental responses, have been designed previously to decrease the gap between machinery and the natural world. Such provoked mechanical components can generate active force and motions in response to applied stimuli such as light,^[^ ^2^ ^]^ temperature,^[^ ^3^ ^]^ humidity,^[^ ^4^ ^]^ magnetic fields,^[^ ^5^ ^]^ electric,^[^ ^6^ ^]^ and pH level^[^ ^7^ ^]^), and make a big difference in the fields like soft robotics, artificial muscles, and biomimetic devices.^[^ ^1c^ ^]^ Besides, the emerging functional materials, like liquid crystal elastomers,^[^ ^8^ ^]^ stimuli‐responsive hydrogels,^[^ ^9^ ^]^ thermal‐responsive polymers,^[^ ^10^ ^]^ conducting polymers,^[^ ^11^ ^]^ dielectric elastomers^[^ ^12^ ^]^ and magnetic composite materials^[^ ^13^ ^]^ have significantly boosted the effectiveness of soft actuators in recent years. The exceptional properties of these materials donate the soft actuators' ability to considerably improve the deformation amplitude, generate large force, accelerate the response time, and execute sequential motion output, making them an ideal choice for widespread application in soft robotics and medical devices.^[^ ^14^ ^]^ However, despite the significant progress in this field, realizing intelligent soft actuators with self‐sensing, real‐time motion feedback, and self‐controlled capabilities remains a huge challenge. Specifically, the most advanced driving strategies currently developed, such as the asymmetric expansion,^[^ ^15^ ^]^ the pneumatic expansion,^[^ ^16^ ^]^ and the hydraulic drive,^[^ ^17^ ^]^ typically rely on materials with purely structural functions that cannot be stimulated by trigger signals. As a result, these approaches cannot sense their own movements, which may yield to hampering their overall functionality.

A traditional strategy for action‐sensing consists of using external cameras and image processing systems to capture the actuation behavior of the soft muscles.^[^ ^18^ ^]^ However, this approach is complex and often immobile. Inspired by biological sensing systems, some researches have showcased soft actuators that incorporate mechanical‐sensing features. Therefore, various sensing devices, based on optical loss,^[^ ^19^ ^]^ capacitance,^[^ ^20^ ^]^ electroluminescence,^[^ ^20^ ^]^ triboelectricity,^[^ ^21^ ^]^ and piezoresistivity,^[^ ^22^ ^]^ were physically laminated or embedded with soft actuators to perform the self‐sensing function. For instance, Metin et al.^[^ ^15a^ ^]^ successfully incorporated flexible microcrack‐based strain sensors onto the pneumatically actuated soft gripper fingers. As a result, the soft gripper fingers were able to accurately discern their touch status, contact force, and bending position. A single elastomer fiber artificial muscle^[^ ^23^ ^]^ was also designed to achieve sensing, signaling, tensile, and torsional actuation by implementing a bi‐sheath buckled carbon nanotube (CNT) skin on an elastomer fiber core. This inventive design enabled the artificial muscle to be actuate and sense using just a single electrical stimulus. In addition, these sensing approaches, when being used in the artificial muscles, involve the application of electric signals, which enables to capture the information relative to the bending and the contractile deformation during actuation. Despite their substantial merits, one potential issue lies in the possibility of damaging the device or separating the interface due to the modulus mismatch between the two components.^[^ ^24^ ^]^ Additionally, few of them could realize closed‐loop control functions to achieve real‐time posture self‐regulation.

Herein, we proposed a LBL assembling strategy to prevents nanointerfacial detachment or slipping associated with moduli mismatch between the sensing layer and the actuating layer, resulting in stable real‐time sensory feedback and self‐regulation capabilities. We employed a bilayer structure and the classical microcrack‐based structure could facilitate their high sensing performance. The thermally responsive liquid crystal elastomer (LCE), with large and reversible deformation, is selected as the action layer; moreover, the MXene with excellent photothermal conversion and a high Young's modulus (≈330 GPa),^[^ ^25^ ^]^ is chosen to realize the photothermal driving and enhance the driving force. In addition, MXene possesses high conductivity and can generate radial cracks and axial micro ridges during action when being combined with the contracted LCE, allowing it to simultaneously serve as a sensing layer for the self‐sensing attitude. Besides, the linear cationic polymer PDDA is selected as a mediator and stabilizer to assemble the MXene with the LCE, weakening their modulus mismatch, and enhancing the interface interaction to achieve stable driving and sensing. Furthermore, the PM‐LCE can perform grasping, traction, and crawling movements by controlling the Near‐Infrared (NIR) laser. It could even achieve wing actuation and closed‐loop controlled motion, demonstrating its potential in the fields of bionic robotics, artificial muscles and the intelligence of soft actuators.

## 2 Results and Discussion

### 2.1 Preparation Process of the Actuation Sensing Integrated PM‐LCE

**Figure 1:**
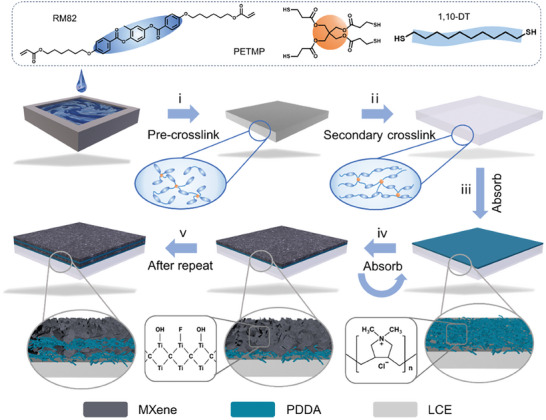
Schematic diagram of the preparation process of PM‐LCE.

**Figure** **1** depicts the preparation process of the PM‐LCE. Firstly, the LCE layer is prepared using Finkelmann's two‐step cross‐linking process. Specifically, the configured LCE monomer solution is prepared, degassed, and injected into a customized poly‐tetra‐fluoro‐ethylene (PTFE) mold to be pre‐crosslinked for ten hours, where 1,4‐bis‐[4‐(6‐acryloyloxyhexyloxy)benzoyl‐oxy]−2‐methylbenzene (RM82) is selected as the liquid crystal monomer, penta‐erythritol tetra (3‐mercapto propionate) (PETMP) as the crosslinker, 1,10‐decane‐di‐thiol (1,10‐DT) as the spacer, 2,2‐di‐methoxy‐2‐phenyl‐acetophenone (DMPA) as the photoinitiator, and di‐propyl‐amine (DPA) as the Michael addition catalyst. Subsequently, the pre‐crosslinked LCE is removed from the mold and cut into the desired shape, then programmed for orientation through mechanical stretching.^[^ ^26^ ^]^ After that, the second crosslinked, under UV irradiation at 365 nm, is conducted to fix the spatial arrangement of liquid crystal primitives to obtain the monodomain elastomers (optimized thickness is 1 mm). Moreover, the delaminated MXene nanosheets are prepared by selectively etching bulk Ti_3_AlC_2_, followed by subsequent exfoliation in distilled water.^[^ ^27^ ^]^ The obtained Ti_3_C_2_T*_x_* nanosheets are predominantly ultrathin nanoflakes with a thickness of ≈1.5 nm and lateral length of 2–3 µm ((Figure S1a,b, Supporting Information). Therefore, these nanosheets confirm that the Ti_3_AlC_2_ MAX is successfully exfoliated into monolayers MXene.^[^ ^28^ ^]^

Moreover, the colloidal suspension of MXene is stable due to its hydrophilicity and electrostatic repulsion^[^ ^29^ ^]^ between neighboring nanospheres, and the Tyndall phenomenon can be observed (as shown in Figure S1c, Supporting Information). A linear cationic polymer PDDA is selected as a mediator and a stabilizer to ensure that the combined PM‐LCE can be actuated without shedding the PM functional layer. Typically, the Ti_3_C_2_T*_x_* MXene nanoflakes are negatively charged whereas the linear cationic polymer PDDA is positively charged (−26.7 mV and +19.9 mV by zeta potential respectively). Impacted by the electrostatic interactions, the Ti_3_C_2_T*_x_* MXene and the PDDA could be assembled layer‐by‐layer (LBL) as a functional layer (denoted as PM layer) on LCE. Specifically, the surface of LCE is treated with oxygen plasma to get a hydrophilic layer (Figure S2, Supporting Information). Then the positively charged PDDA is first adsorbed and soaked in DI water to remove the residual weaker PDDA. Subsequently, the PDDA‐LCE layer is immersed in an aqueous dispersion of monolayer MXene to absorb another layer of negatively charged MXene (step iv). Finally, the above two processes (step iv and v) are repeated until the required layers were achieved.

Besides, to compare the adhesion effects of several assembly processes on the surface of the bilayer membranes, samples are also prepared by applying MXene onto LCE through the methods of spraying and screen printing. All samples prepared by spraying and screen printing exhibit unstable interface adhesion and detachment of the sensing layer. However, the sensing layer and the actuator layer of the PM‐LCE, prepared by the LBL method, are tightly bonded without detachment (Figure S3, Supporting Information). This in situ adsorption assembly strategy allows tight adhesion between the PM functional layer and the LCE layer. In addition, the incorporation of long chains of PDDA moduli alleviates the mismatch between the sensing and the actuating layers and contributes to its flexibility.

### 2.2 Structure and Characterization of PM‐LCE

**Figure 2:**
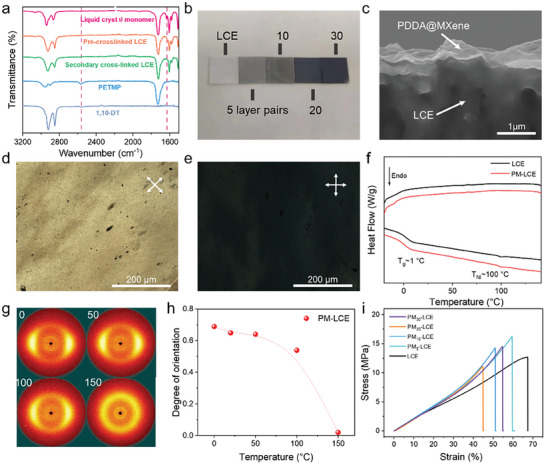
a) FTIR spectra of raw materials and different crosslinking states of LCE. b) Photographs of LCE and PM‐LCE with different thicknesses of PM layers. c) Cross‐sectional SEM images of liquid nitrogen embrittlement of PM_20_‐LCE. POM image of LCE membrane d) before and e) after 45° rotation. f) DSC profiles of LCE and PM‐LCE. g) 2D‐WAXD patterns of PM‐LCE. h) Correlation of orientation and temperature of LCE and PM‐LCE. i) Stress–strain curves of samples perpendicular to the orientation of the LCE and the PM‐LCE.

The structure and morphology of the multilayer coating are further examined. The Fourier Transform Infrared (FTIR) spectroscopy spectrum is characterized to elucidate the synthesis process of LCE. As shown in **Figure** **2**a and Figure S4 (Supporting Information), the ─SH stretching vibration peak of PETMP and 1,10‐DT at 2567 cm^−1^ and the C═C stretching vibration peak in the liquid crystal monomers at 1630 cm^−1^ almost disappear after photopolymerization, demonstrating that the monomers achieve high levels of cross‐linking. The X‐ray diffraction (XRD) pattern (Figure S5, Supporting Information) confirms the successful exfoliation of MAX into MXene.^[^ ^30^ ^]^ In addition, the (002) peaks of the PM showed a significant shift downward from 6.9° to 5.6°, indicating an increase in the spacing between MXene layers upon the inclusion of PDDA. With the adsorption of MXene and PDDA alternately, the samples become successively darker with the number of layer pairs increases from 0 to 30 (Figure 2b). The SEM cross‐sectional images of PM_20_‐LCE showed a distinct bilayer structure. Added to that, there is a direct positive correlation between the number of assembled layers and the thickness of the PM functional layer, and each pair of layers is about 8 nm (as shown in Figure 2c and Figure S6, Supporting Information). Furthermore, clear distribution boundaries of Ti elements in MXene and S elements in LCE could be clearly observed in the EDS images (Figure S7, Supporting Information), further echoing its bilayer structure.

Subsequently, polarized optical microscopy (POM), thermal gravity analysis (TGA), and 2D wide‐angle X‐ray diffraction (2D‐WAXD) are deployed to verify the mesomorphic properties of the above LCE samples. As depicted in Figure S8 (Supporting Information), the pre‐crosslinked LCE exhibits a nematic schlieren texture at 25 °C, and completely turned black at 120 °C, indicating its transition from anisotropy to isotropy while the temperature gets above the clearing point temperature *T*_NI_.^[^ ^31^ ^]^ Moreover, the POM images (Figure 2d,e) of the PM‐LCE show the characteristic changes from dark‐field to bright‐field when being observed between crossed or parallel polarizers, confirming that the LCE in the bilayer PM‐LCE displays uniaxial orientation and exhibits monodomain state.^[^ ^8d^ ^]^ In addition, Figure S9 (Supporting Information) presents the TGA curves of the monodomain samples, and the decomposition temperature (*T*_d_) of LCE and PM‐LCE is approximately 300 °C, indicating that both have good stability. In addition, compared to the pre‐crosslinked and the secondary cross‐linked LCEs, the PM‐LCE exhibits better thermal stability. The phase transition temperature of the samples is then analyzed using the differential scanning calorimetry (DSC) (Figure 2f), the glass transition temperature (*T*_g_) and the clearing point temperature (*T*_NI_) of PM‐LCE appeared at 1 °C and 100 °C respectively, which was consistent with the pure LCE. The 2D wide‐angle X‐ray diffraction (2D‐WAXD) images of LCE film (Figure 2g) further confirm that all the above polymeric materials form a nematic phase. The narrow equatorial diffraction arcs, under room temperature, illustrate the uniaxial orientation of the nematic mesogens in LCE along the mechanical stretch direction.^[^ ^32^ ^]^ When the sample warm up to 150 °C, the 2D‐WAXD pattern shows a uniform ring, indicating its transformation into the isotropic phase under this temperature. Based on the fitting of the azimuthal angle plots, the orientation degree of the sample is calculated using the following equation^[^ ^33^ ^]^:

(1) $$\Pi \;=\frac{180-H}{180}$$

In this equation, Π represents the degree of orientation and *H* corresponds to the half‐maximum width of the azimuthal distribution curve observed in the equatorial reflection. The Π value of PM‐LCE is calculated at about 0.65 under room temperature (Figure 2h), indicating a high‐quality uniaxial orientation of the mesogenic directors inside the LCE samples. Furthermore, a high Π value is beneficial for large deformation under temperature stimulation.

The driving performance of LCEs is greatly influenced by their mechanical properties, so the mechanical measurements of the LCE‐based samples are conducted. As shown in Figure 2i and Figure S10 (Supporting Information), the conventional elastomeric response along with the director (||), are observed in the stress‐strain curves of all monodomain LCE‐based samples. This is in line with the reported elastomeric behavior of the monodomain nematic main‐chain LCEs.^[^ ^34^ ^]^ Moreover, the PM‐LCE exhibits, in both the parallel and perpendicular orientations, a significantly greater tensile strength compared to LCE, which is mainly due to the MXene that has a very high Young's modulus (a single‐layer modulus of around 330 GPa),^[^ ^25^ ^]^ and to the adhesion of the PM layer is very strong.

### 2.3 Mechanical, Photothermal, and Actuating Properties of PM‐LCE

**Figure 3:**
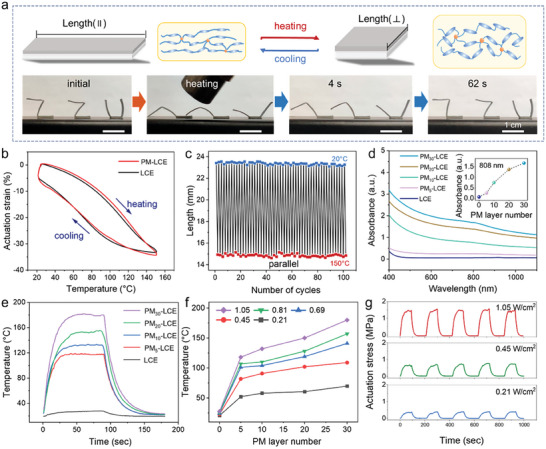
Performance characterization and demonstration of soft actuators based on LCE. a) ZZU model with shape memory based on PM‐LCE. b) Shape memory curves of PM‐LCE. c) Schematic diagram of the variation of film deformation (*L*‐*L*_0_)/*L*_0_ with temperature during the warming and cooling of liquid crystal elastomer film. d) UV–vis spectra of neat LCE and PM‐LCE with 5, 10, 20, and 30 PM layer pairs. e) Heat‐up curves under NIR irradiation of neat LCE and PM‐LCE with 5, 10, 20, and 30 PM layer pairs. f) Temperature of PM‐LCE under NIR light irradiation with different optical power densities. g) Actuating stress of PM‐LCE under laser irradiation with different optical power densities.

LCE is a thermal‐responsive shape memory polymer that deforms with temperature changes.^[^ ^35^ ^]^ As illustrated in **Figure** **3**a, during the cycle process between cooling and heating, both PM‐LCE films and pure LCEs will undergo reversible lengthening and shortening as they experience extension and contraction due to the transition from the liquid crystalline phase to the isotropic phase. Meanwhile, the macroscopic motion of the LCE, such as shrinkage, bending, and rotation, could be programmed by editing the microscopic arrangement of the liquid crystal monomers.^[^ ^36^ ^]^ A soft actuator, having a ZZU shape at room temperature, is tailored by customizing a locally oriented PM‐LCE (Figure S11, Supporting Information). In this process, we folded the prepolymer to induce orientation of the LCE around the folding angle. Further, we performed secondary polymerization by using UV light (365 nm UV lamp for 30 s) to permanently fix this orientation. As depicted in Figure 3a, Figure S11 (Supporting Information), and Video S1 (Supporting Information), the ZZU model is deformed when heated up by a heat air gun and reverted to its original shape when the heat source is removed. During the heating process, the oriented liquid crystal monomers transformed from the liquid crystal phase to the isotropic phase, causing the actuators to flatten into the programmed shape, thereby achieving large‐angle deformation of the actuators. Once colling down, the isotropic phase recovered to the nematic state, resulting in reversible shape change.

To study the thermo‐mechanical properties of LCE and PM‐LCE, a dynamic mechanical analyzer (DMA Q800) is used to investigate the relationship between the sample temperature and the actuation strain. Moreover, DMA's program is utilized to create a temperature‐time curve for the furnace. The initial heating and cooling cycle are implemented to alleviate any internal stress in PM‐LCE material. Therefore, the following heating and cooling cycle data is used to generate a relationship curve connecting temperature and actuation strain (Figure S12, Supporting Information). The strain changes of LCE and PM‐LCE are similar (Figure 3b), indicating that the PM layer did not affect the thermo‐mechanical properties of LCE. When the temperature increases, the PM‐LCE starts to produce actuation strain, and the sample gradually contracts and shortens. The initial strain change rate is relatively low. However, as the heating temperature rapidly increases, the change rate becomes higher and the highest rate occurs in the temperature range of 80–110 °C. Moreover, as the temperature continues to increase and reaches the range of 110–150 °C, the actuation strain gradually increases, but the change rate slowly decreases. The maximum value of 35% is reached at a temperature of 150 °C. Furthermore, Figure 3c and Figure S13 (Supporting Information) showed the changes of length in the long axis parallel to the orientation direction and the short axis perpendicular to the orientation direction. It is clear that, PM‐LCE can still recover the initial shape after undergoing 100 heating–cooling thermal‐mechanical cycles, demonstrating its excellent shape memory stability.

The PM functional layer also provides PM‐LCE with excellent photothermal performance. As shown in the UV‐Vis‐NIR absorption spectrum in Figure 3d, pure LCE presents a negligible absorption in the visible and infrared regions; however, after absorbing the PM layer, the sample exhibited a wide absorption in the 200–1200 nm region with an absorption peak at 808 nm. Furthermore, the maximum absorption increases with the thickness of the PM layers. To assess the photothermal actuation capabilities of the LCE‐based samples, sophisticated tools, including a thermal imager (Testo 869) and specific light sources, such as an 808 nm NIR laser, are deployed. As indicated in Figure 3e, under NIR irradiation, the surface temperature of the pure LCE increases slightly; however, PM_5_‐LCE, PM_10_‐LCE, PM_20_‐LCE and PM_30_‐LCE exhibit significant temperature increases, reaching peaks of 28, 119, 134, 158 and 182°C, respectively. The surface temperature of PM_30_‐LCE reaches a maximum value within 26 seconds. Therefore, Figure 3f shows the temperature curves of PM‐LCE at different optical power densities of NIR irradiation. Referring to this figure, the heating rate is positively correlated with the optical power density and the PM layers. Moreover, the actuation stress, generated by the PM‐LCE, is found to be directly proportional to the PM layers and the optical power density (Figure 3g and Figures S14 and S15, Supporting Information). In more detail, Figure 3g presents the variation of the actuating stress of PM_30_‐LCE with 0.21, 0.45, and 1.05 W cm^−2^ of NIR laser where the maximum actuating stresses can reach 0.38, 0.72, and 1.56 MPa respectively. However, pure LCE exhibited almost no infrared driving ability (Figure S14, Supporting Information). The actuating stress of PM‐LCE far exceeds human muscles (≈0.35 MPa), making it possible for many biomimetic applications. To visually demonstrate the photothermal actuation performance of the PM‐LCE, a light‐driven lifting experiment is carried out. Referring to Figure S16 (Supporting Information), the PM‐LCE film (≈53 mg) could easily lift counterpoises with a hook weight of 52, 102, and 202 g, irradiated by the NIR laser. Finally, the maximum lifting weight is more than 4000 times its own weight (200 g of hook weight and 2 g of metal clips).

### 2.4 Self‐Sensing and Closed‐Loop Control

**Figure 4:**
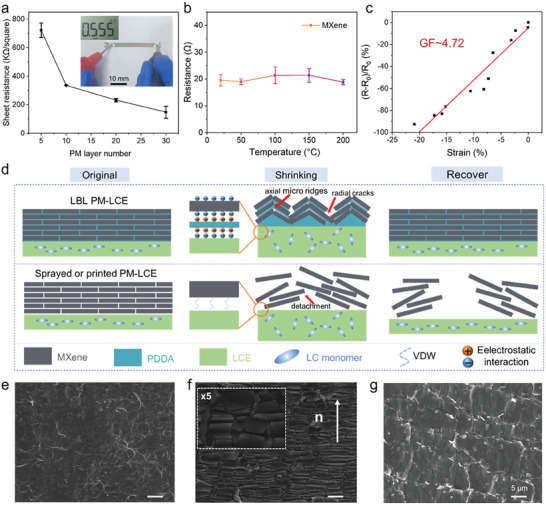
Demonstration of the electrical signal of the PM‐LCE. a) Sheet resistance of PM‐LCE for different thicknesses of PM layers, inset shows the results of PM‐LCE resistance measurements using a multimeter. b) The resistance of MXene as a function of temperature. c) The resistance of the PM‐LCE varies under various strains, GF values can be obtained by linear fitting. d) Schematic representation of the structure of the samples prepared by different processes after deformation. e–g) SEM images of the surface of the PM layer before and after actuation.

The high metallic conductivity (up to 6500 S cm^−1[^ ^37^ ^]^) of MXene endows the prepared PM‐LCE with good conductivity. As displayed in **Figure** **4**a, the sheet resistance of the PM‐LCE film decreases when the PM layers increase. Moreover, PM_30_‐LCE has a sheet resistance of 180 KΩ sq^−1^, which is consistent with the previous reports.^[^ ^29b^ ^]^ Meanwhile, to eliminate the influence of the temperature on the sensor signal, its conductivity is tested at different temperatures. Therefore, Figure 4b shows that the resistance of the MXene can remain relatively stable within 0– 200 °C, allowing it to be used as a sensor under variable temperature conditions. Upon being stimulated by the IR light, the photothermal effect causes a change in the local temperature of the PM‐LCE, leading to shorten (or enlarge) the sample in the orientation direction and deformation in the direction perpendicular to the orientation. Figure 4c exhibited the resistance change as a function of the applied strain for the optimized PM‐LCE sensor where a characteristic linear response in 0–22% regions is observed. This is a novel strain sensing that differs from the traditional microcracks‐based strain sensing, which reduces the conductive path and increases the resistance under strain. The calculated sensitivity values of the Gauge factors (GF) for these regions are 4.7. The linear response of the PM‐LCE sensing film represents the result of uniform formation of the recoverable radial cracks and axial micro ridges across the whole film, which can be restored to a relatively flat state after the removal of light stimulation (Figure 4e–g). Finally, the relative resistance changes of the proposed strain sensor, under cyclic strain, are recorded as shown in Figure S17 (Supporting Information), and the stable response curve shows its good sensing stability. Compared with embedded structure^[^ ^2c^ ^]^ or device that involving complex components,^[^ ^6c^ ^]^ this bilayer‐structured self‐sensing actuator, employed classical microcrack sensing theory to monitor motion, is more simplified and could facilitate higher sensing performance.

The principle of suppressed interface mismatch and stable response is illustrated in Figure 4d. Taking the orientation direction of the liquid crystalline motif as the axis, when the PM‐LCE performs shrink actuation, the PM layer forms recoverable radial cracks and axial micro ridges as shown in Figure 4d,f. This leads to the increase in the charge transport pathways and to the decrease in the resistance. During the deformation process, MXene nanosheets tightly adhere to the LCE layer due to the electrostatic interaction with PDDA. Thus, the radial cracks and the axial micro ridges can recover to their initial state. However, for samples prepared using printing or spraying methods, there is only a weak van der Waals force between MXene and LCE, resulting in detachment due to the interface mismatch after multiple movements (Figure S4, Supporting Information). By using the LBL assembly strategy and the electrostatic interaction, the potential issues, caused by the difference in the moduli between the sensing and actuating layers in smart sensors can be effectively addressed. These strategies work together to prevent the unstable combination and response of the sensors, ensuring therefore an accurate and reliable readings.

**Figure 5:**
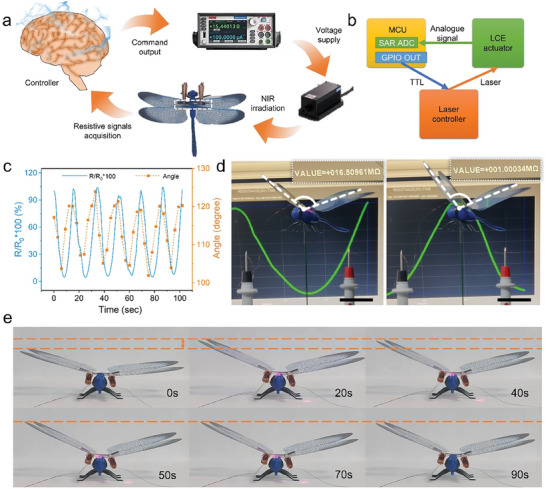
Demonstration of the application of PM‐LCE for self‐sensing. a) Schematic diagram of closed‐loop control. b) Principle of closed‐loop control of PM‐LCE is realized by MCU. c) Relative resistance of PM‐LCE and the angle of dragonfly's wings as a function of time. d) Application demonstration of the light‐driven dragonfly constructed by PM‐LCE. e) Optical images of dragonfly in a stable attitude during closed‐loop control.

Furthermore, the self‐sensing function with closed‐loop control is indispensable in the development of intelligent soft actuators. Inspired by the spontaneous response of human muscles and the octopus tentacles, the PM‐LCE‐based closed‐loop control system is analyzed (**Figure** **5**a). After being irradiated by the NIR light, the PM‐LCE will contract and actuate, and the resistance will change accordingly. The microcontroller unit receives the signal of the resistance change, identify it, and issues a command to modulate the NIR laser irradiation to realize the closed‐loop control of the PM‐LCE actuation (Figure 5b). The light‐driven dragonflies are assembled to explore the self‐sensing and closed‐loop control functions. When stimulated by a NIR laser, the PM‐LCE is heated to drive the dragonfly's wings (Figure 5c). After several preactivated deformations, the PM layer can reach a stable stage without significant detachment to form stable microcracks. By connecting wires at both ends, the resistance signal of the PM‐LCE motion can be monitored in real‐time. Meanwhile, the PM‐LCE can adapt to feedback changes in the state of motion through resistance signals. The correspondence between the bending angle and the resistance during PM‐LCE bending are shown in Figure 5d. Once a fixed angle command is inserted, the closed‐loop control system identifies the existing position through resistance and achieves specific position adjustments by adjusting the light source (Figure 5e and Video S2, Supporting Information). The establishment of this closed‐loop control system can offer valuable insight for precise control and equipment intelligence in the future.

## 3 Conclusion

In this paper, an integrated strategy to prepare NIR‐driven and self‐sensing intelligent bilayer soft actuator PM‐LCE was presented. The high‐precision, self‐sensing and feedback loop control functions actuator were realized by combining the actuation and sensing materials into a bilayer membrane through the LBL self‐assembly process. This assembly process prevents potential detachment associated with the moduli mismatch between the sensing layer and the actuating layer interface and exhibits stable interface adhesion and with tightly bonded. Due to the photothermal effects and the high conductivity, the prepared PM‐LCE could simultaneously respond to the light and sense its own motion state. The grasping, traction and crawling movements could be performed with the control of the NIR laser. Finally, the PM‐LCE‐based closed‐loop control system was also developed and demonstrated, which was beneficial for precise control and equipment intelligence in the future. To sum up, the current research provides a new strategy for developing soft actuators integrated with self‐sensing and actuation functions and demonstrates its potential in the fields of bionic robotics, artificial muscles and the intelligence of soft actuators.

## Conflict of Interest

The authors declare no conflict of interest.

## Supporting information

Supporting Information

Supplemental Video 1

Supplemental Video 2

## Data Availability

The data that support the findings of this study are available from the corresponding author upon reasonable request.
