# Supplementary material for: Near‐Infrared Light‐Driven MXene/Liquid Crystal Elastomer Bimorph Membranes for Closed‐Loop Controlled Self‐Sensing Bionic Robots
Source: Adv Sci (Weinh). 2023 Nov 20;11(2):2307862. doi: 10.1002/advs.202307862 (PMC10787073; doi:10.1002/advs.202307862)
Supplement: Supplementary file 1 — Supporting Information [file ADVS-11-2307862-s001.pdf]

ADVANCED SCIENCE
Open Access

Supporting Information

for *Adv. Sci.*, DOI 10.1002/advs.202307862

Near-Infrared Light-Driven MXene/Liquid Crystal Elastomer Bimorph Membranes for Closed-Loop Controlled Self-Sensing Bionic Robots

Youwei Yang, Lingxian Meng, Juzhong Zhang, Yadong Gao, Zijuan Hao, Yang Liu, Mingjun Niu, Xiaomeng Zhang, Xuying Liu* and Shuiren Liu**

Supporting Information

Near-Infrared Light-Drived MXene/Liquid Crystal Elastomer Bimorph Membranes for Closed-Loop Controlled Self-Sensing Bionic Robots

Youwei Yang+, Lingxian Meng+, Juzhong Zhang+, Yadong Gao, Zijuan Hao, Yang Liu, Mingjun Niu, Xiaomeng Zhang, Xuying Liu* and Shuiren Liu**

Y. Yang, L. Meng, J. Zhang, Y. Gao, Z. Hao, Prof. M. Niu, Prof. X. Zhang, Prof. X. Liu, Prof. S. Liu

School of Materials Science and Engineering, Zhengzhou University, Zhengzhou, 450001, People's Republic of China.

E-mail: liuxy@zzu.edu.cn; niumj@zzu.edu.cn; lsrzzdx@zzu.edu.cn.

Y. Liu

School of Chemical Engineering, Zhengzhou University, Zhengzhou, 450001, People's Republic of China.

[+] The authors are equally contributing to this work.

Experimental Procedures

Materials: 1,4-bis-[4-(6-acryloyloxyhexyloxy)benzoyl-oxy]-2-methylbenzene (RM82, 95%), was purchased from HEOWNS. Pentaerythritol tetra(3-mercaptopropionate) (PETMP, 95%), 1,10-decanedithiol (1,10-DT, 96%), was obtained from Macklin. 2,2-dimethoxy-2-phenylacetophenone (DMPA, 99%), dipropylamine (DPA, 99%), Poly dimethyl diallyl ammonium chloride (PDDA) were obtained from Aladdin. All materials were used as received unless otherwise noted.

Preparation of LCE: RM82 (1.1 g, 0.00164 mol), PETMP (0.0726 g, 0.00015 mol), DT (0.276 g, 0.00134 mol) and DMPA (0.0645 g, 0.0025 mol) were dissolved in toluene, then 1% DPA in toluene was added as the Michael addition reaction catalyst. Pre-crosslinking is completed by heating in an oven at 80 °C for 12 hours, followed by 24 hours under vacuum to evaporate the solvent. After demolding, uniaxially stretched to 200% and subjected to 365 nm UV light for 20 min to accomplish secondary crosslinking.

Preparation of $Ti_3C_2T_x$ nanosheets: Firstly, 2 g LiF was weighed and added to the Teflon reaction flask. After the magnetic stirrer was put in, 30 ml hydrochloric acid and 10 ml ultrapure water were injected, stirring for 30 min, and then 2 g Ti_3AlC_2 MAX phase was slowly added within 5 min. Then the reaction flask was placed in a water bath at 35 °C for 24 hours for etching. The suspension obtained after etching was put into a centrifuge tube, and ultra-pure water was added to put the centrifuge tube into an ultrasonic cleaning instrument for 5 min. Then a high-speed centrifuge was used to centrifuge at 3500 rpm for 5 min to measure the pH value of the supernatant, and the above process was repeated until the pH value was about 5. Then, the $Ti_3C_2T_x$ MXene was layered into $Ti_3C_2T_x$ MXene nanosheets by intercalation with absolute ethanol and shaking. Finally, ultra-pure water was added to low-speed centrifugation, and the upper suspension was $Ti_3C_2T_x$ MXene nanosheet dispersion.

Preparation of PM-LCE: Firstly, the liquid crystal elastomer surface, which has been fixed orientation, was treated using oxygen plasma cleaning. Then, uniformly dispersed aqueous solution of $Ti_3C_2T_x$ MXene (1 mg/mL) and aqueous solution of PDDA (1 mg/mL) were prepared, respectively. The liquid crystal elastomer was fixed on the glass substrate and immersed in the PDDA aqueous solution for adsorption, and then washed with ultra-pure water to adsorb the unstable PDDA on the surface and air dried at room temperature to complete the

first layer assembly. Then the liquid crystal elastomer was immersed in $Ti_3C_2T_x$ MXene aqueous dispersion for a second layer of electrostatic self-assembly, also washed with ultra-pure water and air dried. The above two adsorptions were taken as a group, and the preparation of PM-LCE could be completed by repeating the above operations for PM alternating lamellar self-assembly (LBL).

Fabrication of "ZZU" actuator based on PM-LCE: To achieve large-angle bending of liquid crystal elastomer actuators, we used "origami" method to program the material. As shown in Figure S11, the pre-crosslinked LCE was cut into 30 × 10 mm shapes and placed on a mold to form the shape of "ZZU". In this process, we folded the prepolymer to induce orientation of the liquid crystal monomers around the folding angle. Further, we performed secondary polymerization by using UV light (365 nm UV lamp for 20 min) to permanently fix this orientation in the liquid crystal elastomer actuators. Finally, the PM layer was added by LBL.

Fabrication of PM-LCE-based closed-loop control system: Pre-crosslinking of LCE is completed by heating in an oven at 80 °C for 12 hours, followed by 24 hours under vacuum to evaporate the solvent. After demolding, uniaxially stretching to 200% was employed and then subjected to 365 nm UV light for 20 min to accomplish secondary crosslinking. Finally, the PM-LCE was obtained by LBL. The uniaxial oriented PM-LCE was cut into long strips and pasted on the back of the dragonfly models (commercially available), and the two ends of the PM-LCE were fixed to each wing using polyimide tape. The real-time resistance was measured by a digit multimeter (DMM 6500). The MCU (ESP8266) with a 10 bit ADC module was programmed for the closed-loop control of this system. The microcontroller unit receives the signal of the resistance change, identify it, and issues a command to modulate the NIR laser irradiation to realize the closed-loop control of the PM-LCE actuation.

Characterization: Fourier transform infrared spectrometer (Bruker, Tensor-27) was used to characterize the samples. The sample was tested using an X-ray diffractometer (Rigaku, SmartLab SE) in a BB mode. The samples were tested using a differential scanning calorimeter (TA, DSC25) in a lift-fall-lift manner, with the first heating removing the thermal history and the second heating and first cooling for data analysis. Scanning electron microscope (Tescan, MIRA LMS), transmission electron microscope (Thermo fisher, Talos F200S) and atomic force microscope (Bruker, Nanowizard 4XP) were used to observe the morphology of the samples.

The relationship between liquid crystal phase and orientation of samples with temperature was studied by using 2D wide Angle X-ray diffractometer (Bruker, D8 Discover). Optical images and POM images of the samples were obtained using a polarizing microscope (Leica, DM2700M). In combination with the 808 nm NIR laser (LASEVER, LSR808H-8W-FC), actuation stress of the sample was measured by the universal material testing machine (Shimadzu, AGS-X), and the temperature change of the sample was recorded by the infrared thermal imager (Testo, 869).

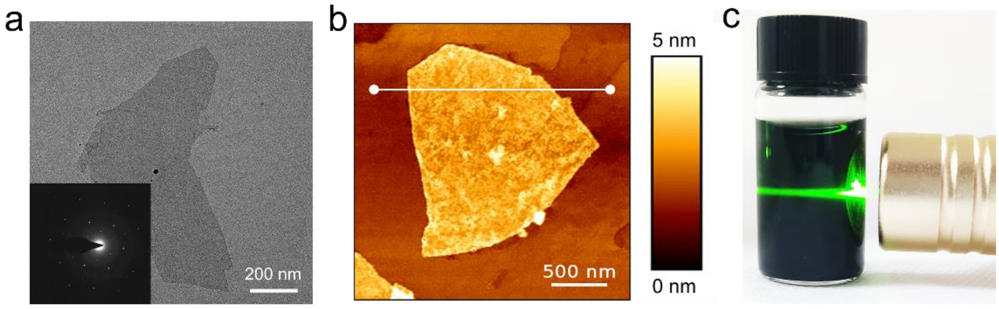

Figure S1. a) TEM image of a $Ti_3C_2T_x$ MXene nanoflake, inset is the selected-area electron diffraction (SAED) of $Ti_3C_2T_x$ MXene. b) AFM image of a $Ti_3C_2T_x$ MXene nanoflake. c) Tindal effect of MXene aqueous dispersions, suggests good dispersion of MXene in water.

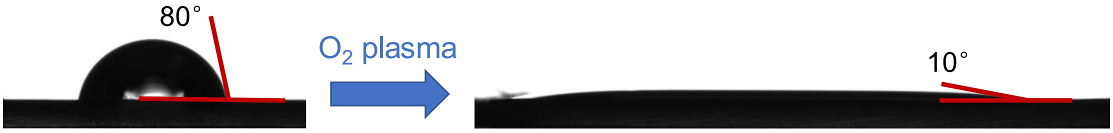

Figure S2. Water contact angles of the surface of LCE in different states

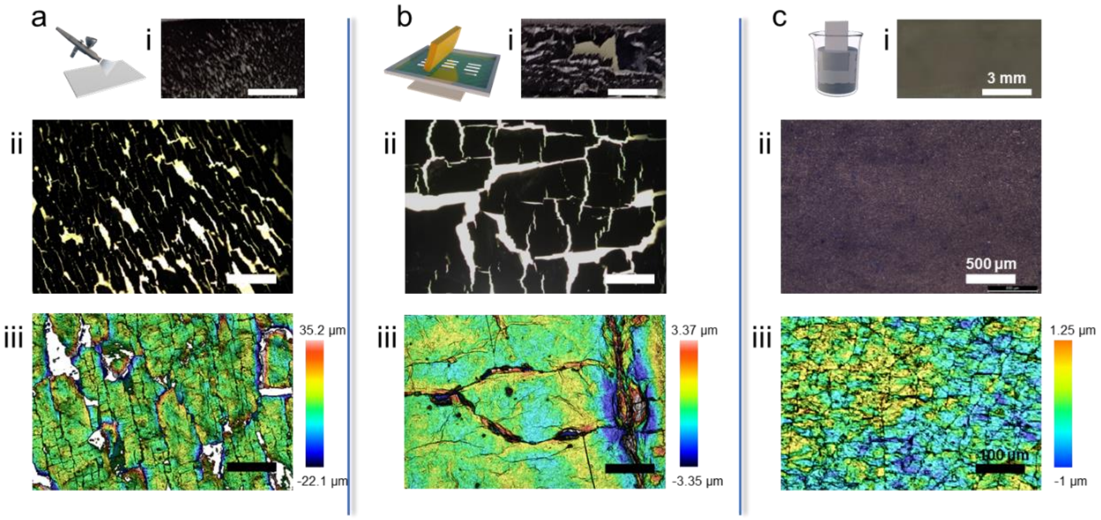

Figure S3. i)Photographs, ii) microscope images and iii) 3D optical profilometer images of samples prepared by a) inkjet printing, b) screen printing and c) LBL after deformation.

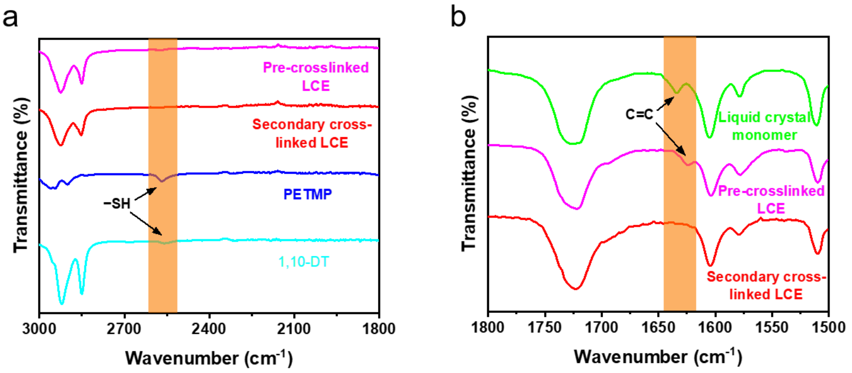

Figure S4. Local magnification of the FT-IR spectra of LCE: a) -SH; b) C=C.

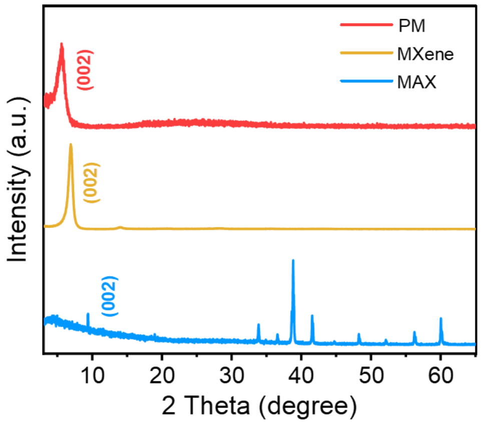

Figure S5. XRD patterns of MAX, MXene, and PM.

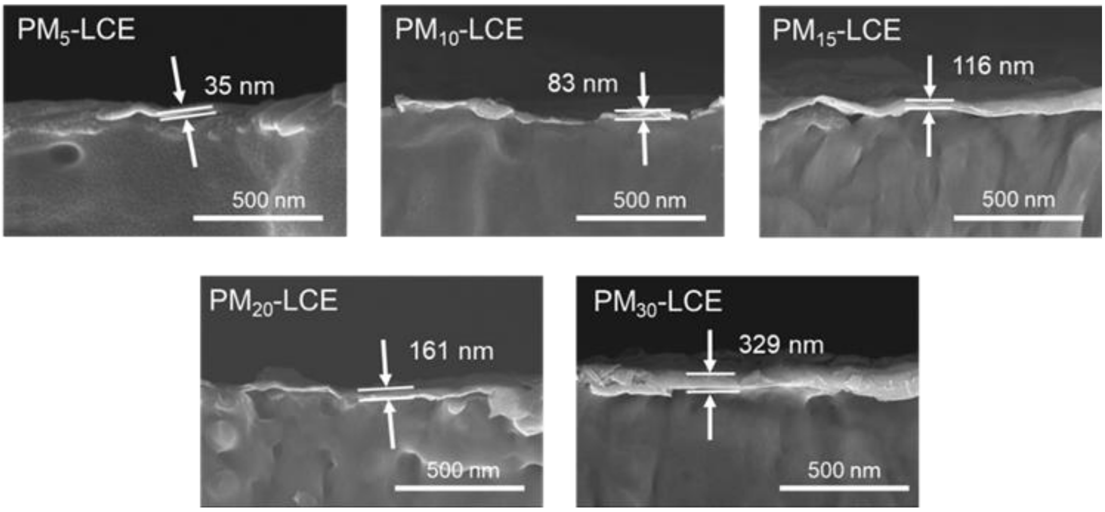

Figure S6. SEM cross-sectional images of PM-LCE for 5, 10, 15, 20, 30 layer pairs reflecting different thicknesses.

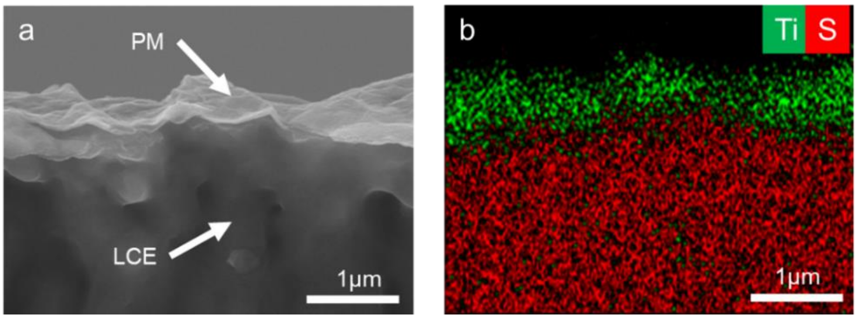

Figure S7. a) Cross-sectional SEM image of PM_{20}-LCE b) An EDX image depicting the distribution of cross-sectional Ti, S elements of PM_{20}-LCE.

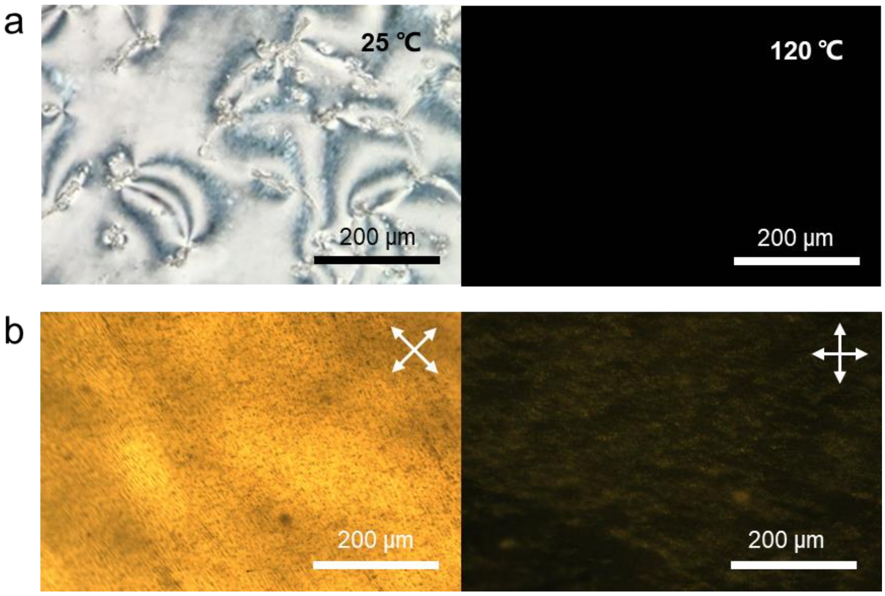

Figure S8. a) Precrosslinked LCE polarizing microscope images at 25℃ and 120℃; b) Birefringence of LCE in uniaxial orientation.

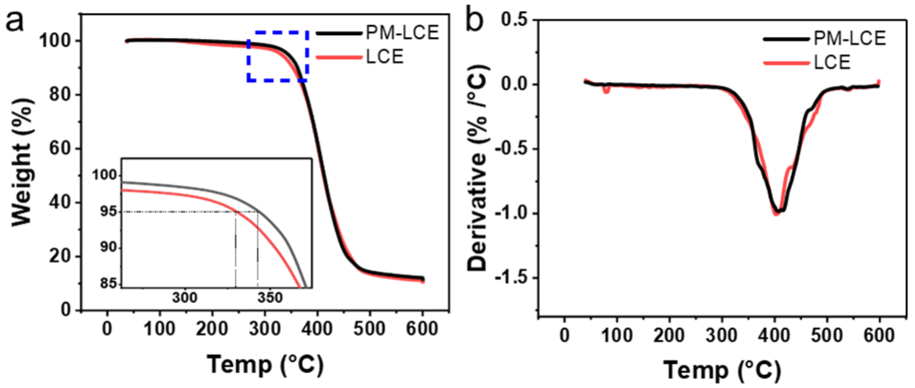

Figure S9. Comparison of a) TGA and b) DTG curves of LCE show the good thermal stability of LCE.

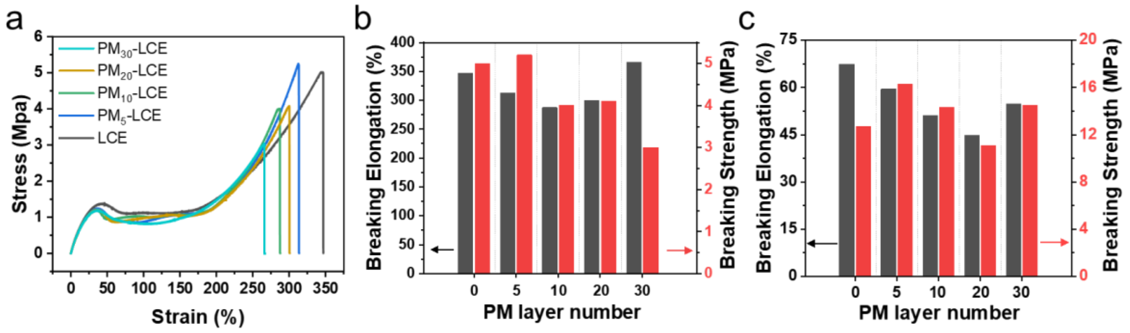

Figure S10. Stress-strain curves a) perpendicular to LCE and PM-LCE orientations. Fracture strength and strain b) perpendicular to and c) parallel to LCE and PM-LCE orientations.

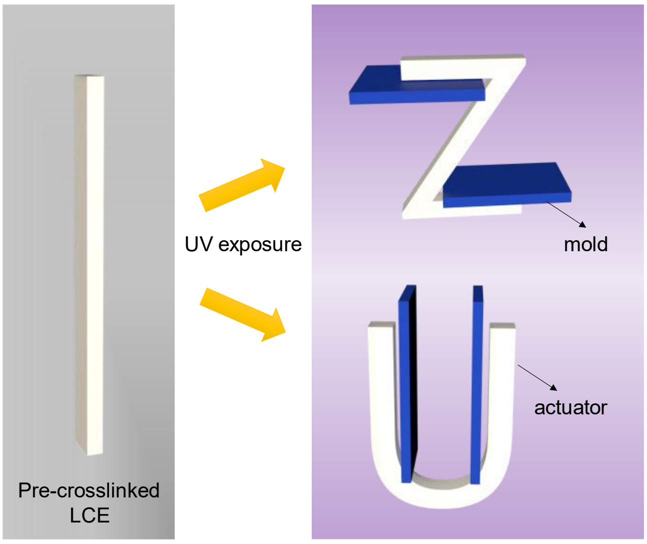

Figure S11. Schematic diagram of the preparation of "ZZU" actuator.

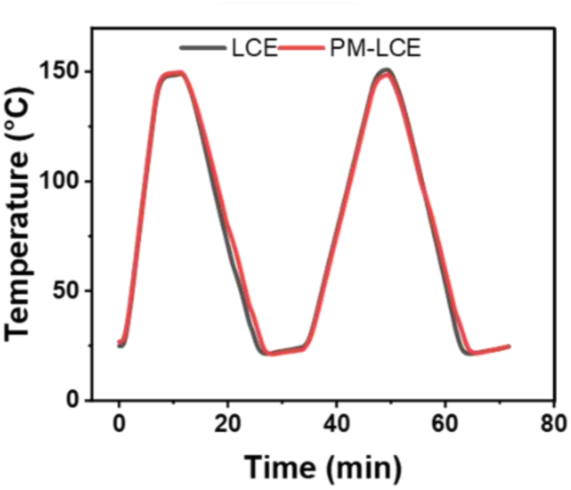

Figure S12. Curve of temperature during DMA testing.

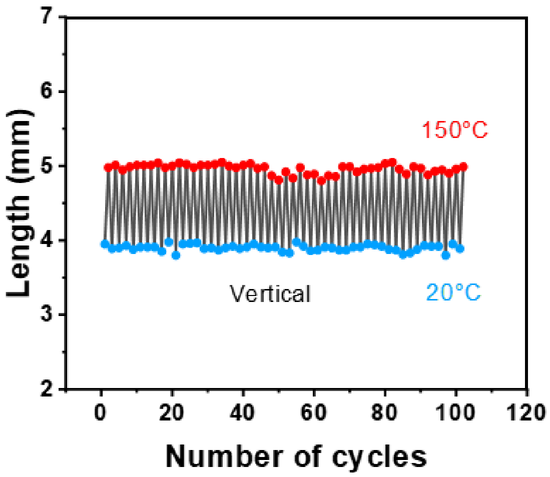

Figure S13. Schematic diagram of the deformation (L-L0) /L0 of the short axis of the film as a function of temperature during the heating and cooling process of the liquid crystal elastic film.

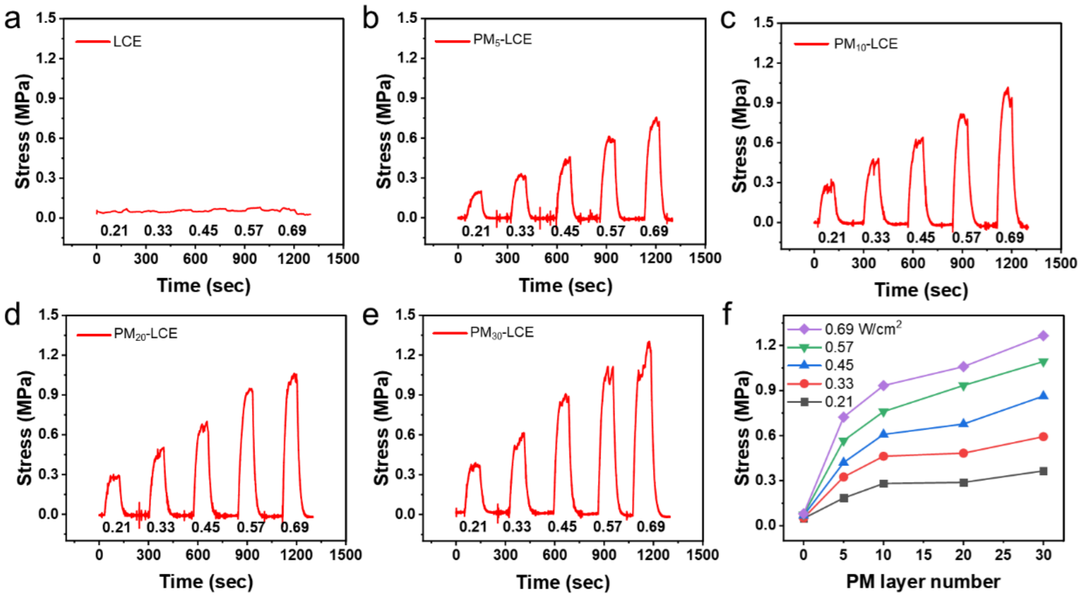

Figure S14. Actuation stress of LCE and PM-LCE under NIR laser irradiation with different optical power densities (W/cm^2).

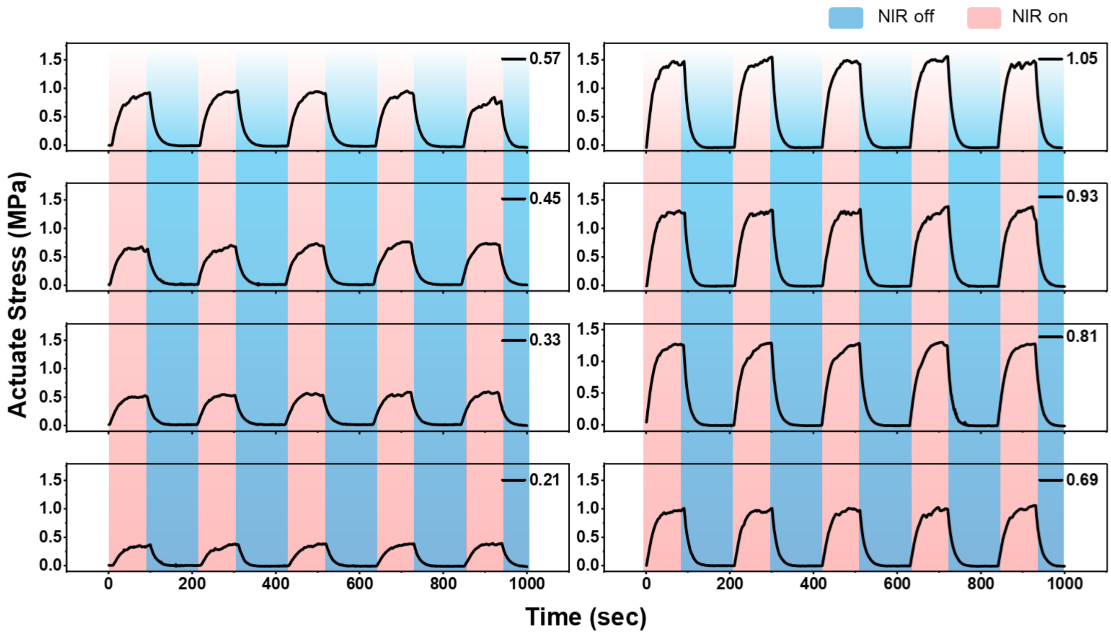

Figure S15. Actuation stress of PM_{20}-LCE under intermittent irradiation of NIR light with different optical power densities (W/cm^2).

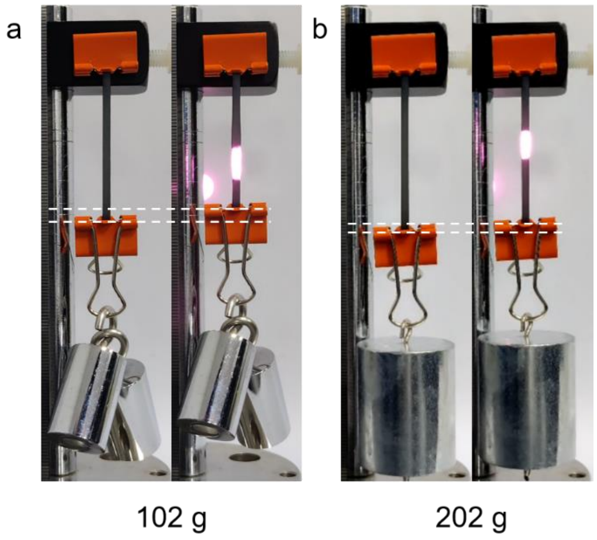

Figure S16. Demonstration of PM-LCE lifting heavy objects during NIR laser irradiation (1.05 W/cm^2).

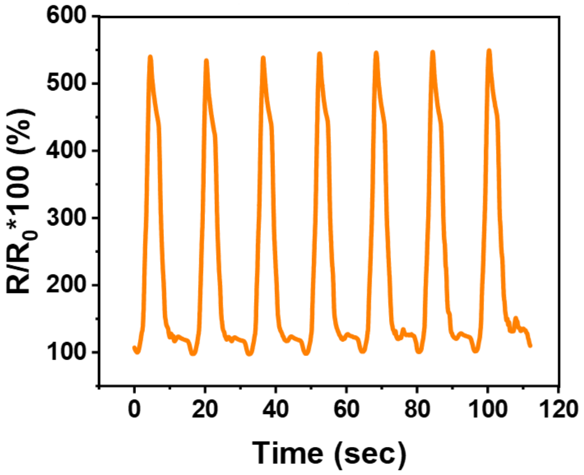

Figure S17. Relative resistance changes of PM-LCE under cyclic strain.
